# WormNet: A Multi-View Network for Silkworm Re-Identification

**DOI:** 10.3390/ani15142011

**Published:** 2025-07-08

**Authors:** Hongkang Shi, Minghui Zhu, Linbo Li, Yong Ma, Jianmei Wu, Jianfei Zhang, Junfeng Gao

**Affiliations:** 1 Sericultural Research Institute, Sichuan Academy of Agricultural Sciences, Nanchong 637000, China; 2 Department of Computer Science, University of Aberdeen, Aberdeen AB24 3FX, UK

**Keywords:** insect re-identification, multi-order extraction, channel interaction, spatial purification, silkworm

## Abstract

Re-identification (ReID) has been widely applied in person and vehicle recognition tasks. This study extends its application to a novel domain: insect (silkworm) recognition. However, unlike person or vehicle ReID, silkworm ReID presents unique challenges, such as the high similarity between individuals, arbitrary poses, and significant background noise. To address these challenges, we propose a multi-view network for silkworm ReID, called WormNet, which is built upon an innovative strategy termed extraction purification extraction interaction. Specifically, we introduce a multi-order feature extraction module that captures a wide range of fine-grained features by utilizing convolutional kernels of varying sizes and parallel cardinality, effectively mitigating issues of high individual similarity and diverse poses. Next, a feature mask module (FMM) is employed to purify the features in the spatial domain, thereby reducing the impact of background interference. To further enhance the data representation capabilities of the network, we propose a channel interaction module (CIM), which combines an efficient channel attention network with global response normalization (GRN) in parallel to recalibrate features, enabling the network to learn crucial information at both the local and global scales. Additionally, we introduce a new silkworm ReID dataset for network training and evaluation. The experimental results demonstrate that WormNet achieves an mAP value of 54.8% and a rank-1 value of 91.4% on the dataset, surpassing both state-of-the-art and related networks. This study offers a valuable reference for ReID in insects and other organisms.

## Simple Summary

In this study, we explore the application of artificial intelligence in silkworm farming by integrating re-identification (ReID) techniques with individual silkworm recognition and propose a novel silkworm ReID method. To address the challenges posed by arbitrary silkworm postures, high inter-individual similarity, and complex image backgrounds—which hinder the performance of existing pedestrian or object ReID models on silkworm datasets—a specialized network architecture is developed. This architecture incorporates multi-order feature extraction, spatial purification, and channel interaction mechanisms to enhance the network’s performance in identifying individual silkworms. The experimental results demonstrate that the proposed network outperforms baseline networks, attention-based models, and state-of-the-art person ReID approaches. This research provides a valuable reference for individual identification in animals and insects.

## 1. Introduction

Re-identification (ReID) is a pivotal branch of computer vision and deep learning, aimed at identifying images (or groups of images) that share the same identity attributes as a given query image from a set of images or videos. This sophisticated technology has a broad range of applications. For instance, person ReID has facilitated advancements in fields such as surveillance, interaction, and activity recognition [1], while vehicle ReID has significantly contributed to the development of intelligent transportation systems [2]. Leveraging the potential advantages of this technology and its application in other domains, such as biology and entomology, can not only aid in understanding insect behaviors in a manner akin to human behavior analysis but also offer novel insights into biological research, thus fostering the growth of both fields.

The silkworm (Bombyx mori), which originated in China, holds considerable economic and ecological importance, primarily for its role in protein production and the generation of natural silk. This insect is extensively farmed in China, India, and Southeast Asia [3]. Understanding silkworm behavior is crucial for breeding high-yield varieties and assessing their health status [4]. Similarly to person ReID, silkworm ReID is a valuable tool for behavior analysis. However, unlike pedestrians, who typically maintain a standing posture and exhibit notable variations in appearance, the posture of silkworms is arbitrary in real-world conditions, and their visual similarity is remarkably high. These challenges, compounded by substantial background interference, pose significant hurdles for ReID methods. Current ReID approaches designed for humans or objects are not suitable for silkworms, underscoring the necessity for specialized networks that can achieve more accurate recognition, thereby providing a foundation for future work.

To address this, we propose a multi-view network for silkworm ReID, named WormNet, which is built upon an innovative strategy known as extraction purification extraction interaction. This strategy is supported by three effective modules. To the best of our knowledge, this is the first study to apply ReID technology to the field of insect science, particularly in silkworm recognition.

**Figure 1:**
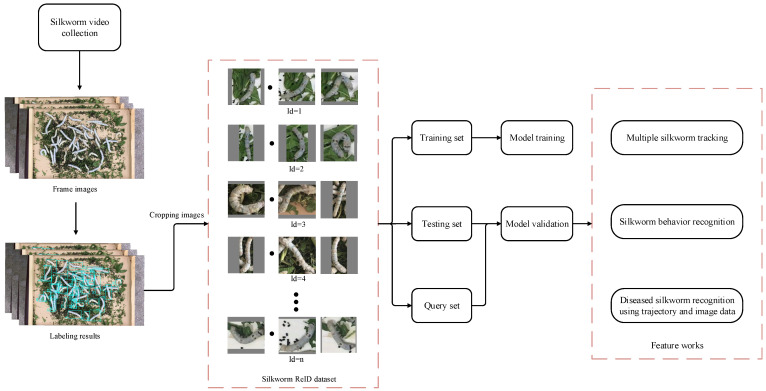
The main steps of silkworm ReID.

Figure 1 illustrates the main process of silkworm ReID. First, videos of silkworm growth are collected, and they are divided into consecutive frames. Then, using an annotation tool, each silkworm is manually tracked from the initial frame to the final frame. Based on the annotated location information, each silkworm is cropped from the image to construct the silkworm ReID image dataset, which is then divided into training, testing, and query sets, used for model training and validation, respectively. Finally, the trained model is used for tracking multiple silkworms, behavior recognition, and the identification of diseased silkworms based on the combined image and trajectory data.

In summary, the main contributions of this paper are as follows:

(1) The introduction of ReID technology use in insect science, specifically for individual silkworms, which can provide a new method for health status assessment and behavior recognition.
(2) The proposal of a multi-view network based on a novel strategy called extraction purification extraction interaction, with three effective modules designed to support this approach.
(3) The proposed network achieves an mAP of 54.8% and a rank-1 of 91.4% on the dataset, outperforming both related and state-of-the-art networks.

The structure of this paper is as follows: In Section 2, we introduce related work, including the current research on person ReID and feature interaction networks. In Section 3, we present the proposed network. In Section 4, we cover the dataset, comparative experiments, and the analysis and discussion of the results. Finally, in Section 5, we provide the conclusion and outline future research directions.

## 2. Related Work

### 2.1. Current ReID Studies

In recent years, deep learning-based ReID research has achieved significant advancements, with numerous networks being introduced. Luo et al. utilized ResNet-50 as a baseline and incorporated a set of training techniques to develop a person ReID model [5]. Zhou et al. proposed an adaptive sparse pairwise loss method that utilizes a select few relevant pairs to enhance the identification performance [6]. Chen et al. introduced an attention-based feature learning approach for person ReID, employing global attention to mitigate background influence and local attention to decouple features that are specifically responsible for different body parts [7]. Baisa designed a multi-branch network that integrates spatial attention, channel attention, and both global and local feature representations to learn more discriminative information [8]. An unsupervised person ReID method was proposed by Sun and Ma, which employs two effective modules to assign and provide reliable feature representations [9]. Gu et al. applied a neural architecture search to person ReID and proposed a twin contrastive mechanism to guide the search process. They also designed a multi-scale interaction search space to optimize the interaction operations between multi-scale features [10]. Liu et al. introduced a cross-modality person ReID network that utilizes parameter sharing between a two-stream network and hetero-center triplet loss to relax the strict constraints of traditional triplet loss [11]. Vision Transformer has also been applied to pedestrian ReID by He et al., who proposed a jigsaw patch module to rearrange patch embedding via shifting and patch shuffle operations [12]. They also introduced side information embedding to alleviate feature bias caused by view variations. Chen et al. proposed a hybrid network for discriminative feature extraction by bridging convolutional and Transformer blocks [13]. Ren et al. developed an occluded ReID framework that leverages obstacle attributes to address occlusion challenges [14]. Xiong et al. presented a clothing-change person ReID network, which incorporates feature attention and reconstruction modules to enhance the network’s ability to process non-clothing features [15].

In this study, we propose a network for silkworm ReID based on several baseline networks. To achieve accurate recognition, we introduce a novel strategy termed extraction purification extraction interaction, with three extraction and interaction modules designed to support this approach.

### 2.2. Feature Interaction in CNNs

Feature interaction is a fundamental technique in convolutional neural networks (CNNs) that enhances the model’s capacity to capture both local and global feature dependencies. Recent studies have demonstrated that incorporating feature interaction mechanisms, such as channel and spatial attention [16], significantly improves the performance in tasks involving complex object recognition and scene understanding. Ouyang et al. designed an efficient multi-scale attention module to facilitate feature interaction within each feature group across channel dimensions [17]. Li et al. introduced cross-stage feature interaction to enhance the backbone features of each stage by incorporating information from adjacent stages for camouflaged object detection [18]. Cai et al. utilized two depth-wise strip convolutions to extract object features at varying scales and capture the local context effectively [19]. Fu et al. proposed a feature interaction module to eliminate modality-specific emotional differences using contrastive losses for multi-modal emotion recognition [20]. Zhang and Yang aimed to capture pixel-level pairwise relationships by grouping channel dimensions into multiple sub-features, utilizing a shuffle unit to model both spatial and channel dependencies [21]. Li et al. introduced an interaction information method for drug–drug interaction prediction, employing a contrastive learning network with edge-aware augmentations and mutual information estimators [22]. Li et al. proposed spatial and channel reconstruction techniques to reduce redundant computation and enhance feature representation learning [23]. Xing et al. introduced a self-attention interaction module for RGB-T tracking, which highlights the fusion of features between RGB and thermal infrared images, facilitating rapid convergence for target localization [24]. Ma et al. developed a graph encoder–decoder convolution module to enable interaction between global visual features in scene understanding tasks [25]. Wan et al. defined a high-order interaction feature selection algorithm by exploring the robust fuzzy joint information shared between two features for classification [26]. Yang et al. combined feature interaction with two pooling styles to enhance the global connectivity of local features in intra-modality person ReID [27]. Li et al. proposed a global feature interaction module using pure convolutional operations to preserve feature information between the encoder and decoder in image segmentation [28].

These studies present a diverse range of feature interaction methods, demonstrating the significant role of this technique in improving model performance across various domains. In this study, we propose two feature interaction modules designed to enhance feature representation in both the spatial and channel dimensions.

## 3. Method

**Figure 2:**
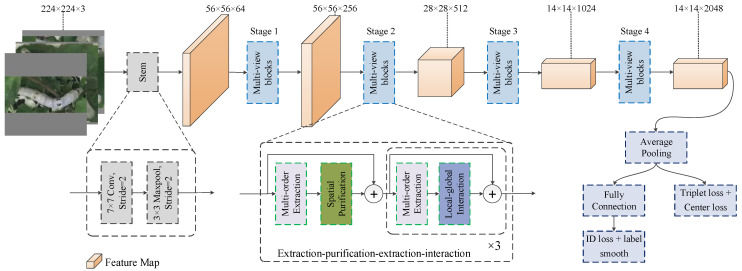
Overview of the proposed network.

In this study, we propose WormNet for silkworm ReID. As illustrated in Figure 2, WormNet is based on a novel strategy termed extraction purification extraction interaction. The first stage, “extraction”, involves feature learning through a multi-order module, the structure of which will be introduced in Section 3.1. At this stage, the extracted features are rough and contain significant interference information. Therefore, “purification” is performed, which refers to the application of a spatial purification module to filter features in the spatial domain, effectively suppressing the background noise. Its structure will be introduced in Section 3.2. Following this, the multi-order feature extraction network is applied again to refine the features, thereby enriching the feature representation and deepening the network’s capacity. Finally, the “interaction” step is undertaken, which refers to the use of a channel interaction module that integrates information at different granularities to achieve a precise characterization of the silkworm’s image. Its structure will be introduced in Section 3.3. The depth is increased by stacking the extraction and interaction modules, where “×3” indicates that stacking is repeated three times, also representing the network’s stage ratio. The proposed strategy and modules are designed to acquire accurate and detailed features.

In the following sections, we will introduce the specific structure (Section 3.4) and loss function (Section 3.5) of WormNet.

### 3.1. Multi-Order Module for Feature Extraction

Feature extraction, or data representation, is the most fundamental aspect of deep learning networks and directly influences their recognition performance. While most current networks utilize convolutional operations or self-attention mechanisms to extract image features, an effective and well-designed structure enhances the network’s discriminative capabilities, thereby ensuring both training efficiency and accurate inference results. To design a silkworm ReID network that balances operational efficiency and a competitive recognition accuracy, we introduce a multi-order feature extraction network based on ResNet [29].

**Figure 3:**
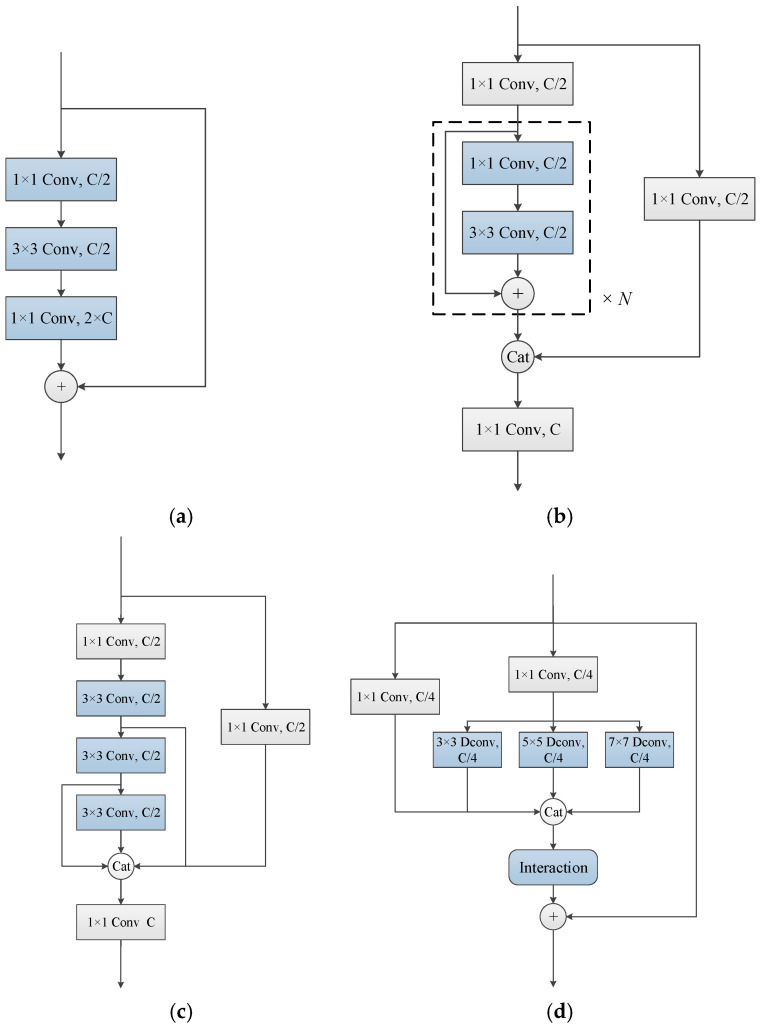
Basic structure of three related modules and our own multi-view module, where ‘Conv’ represents a convolution layer followed by batch normalization and ReLU activation layers, ‘+’ indicates pixel-wise addition, ‘Cat’ denotes feature concatenation, ‘C’ refers to the number of channels, and ‘×N’ indicates N times stacked operations. ‘Dconv’ denotes a depth-wise separable convolution layer, while ‘Interaction’ refers to the feature mask or channel interaction modules proposed in our study. (**a**) Bottleneck of ResNet. (**b**) CSP block. (**c**) E-ELAN block. (**d**) Multi-view block.

Figure 3 illustrates the basic structure of three related modules and our own multi-view modules. ResNet uses 1 × 1 and 3 × 3 convolutional layers to design a bottleneck for feature extraction, and incorporates residual connections to prevent overfitting and mitigate the vanishing gradient problem. The CSP block [30], built upon ResNet, transforms the feature map of the base layer into two branches via 1 × 1 convolution, which are then merged through a cross-stage hierarchy. One branch conducts feature extraction using a bottleneck, while the other serves as a residual connection. E-ELAN [31] further partitions the features of the input layer and applies group convolution to expand both the channels and cardinality of the computational parameters, enabling the network to learn and converge effectively by controlling both the shortest and longest gradient paths. Our multi-view module inherits the residual connection from ResNet and the feature partition from CSPNet to prevent overfitting and enhance computational efficiency. Moreover, we improve the group convolutions by stacking cardinalities into a parallel structure for more effective feature extraction. Standard 3 × 3 convolutional layers are upgraded to 3 × 3, 5 × 5, and 7 × 7 convolutions to capture features with diverse granularities, while depth-wise separable convolutions (dw-conv) are employed to reduce the number of parameters. This approach is referred to as multi-order extraction. A concatenation-based fusion method is also utilized to combine the learned features, maximizing the use of multi-scale convolutional kernels and parallel cardinality to capture fine-grained feature details and enhance the overall feature representation capability.

In practice, when using our multi-view module to extract image features, the input X, with dimensions W×H×C, first undergoes two 1 × 1 convolution operations for channel aggregation and parameter reduction. Notably, the input feature is reshaped to 1/4 of the channel, rather than 1/2, as in CSPNet and E-ELAN. This modification is due to our use of parallel group convolutions; if the feature was transformed to 1/2 of the channel, an additional 1 × 1 convolution would be necessary to ensure that the output and input features maintain the same number of channels after concatenation. We empirically find that over-compressing the channels using a 1 × 1 convolution results in the loss of useful information, which can degrade the generalization performance of the network. As a result, two sub-feature maps, $X_{1}$ and $X_{2}$, with dimensions $W\times H\times \frac{C}{4}$, are obtained through channel aggregation. The formulas for this operation are as follows:

(1) $$X_{1}={Conv}_{1\times 1}(X)$$

(2) $$X_{2}={Conv}_{1\times 1}(X)$$

where ${Conv}_{1\times 1}$ refers to a 1 × 1 convolutional operation.

For one of the sub-feature maps, depth-wise separable convolution (dw-conv) operations with kernel sizes of 3 × 3, 5 × 5, and 7 × 7 are applied separately to learn feature maps at different granularities. Batch normalization (BN) and ReLU activation operations are applied after each convolutional layer. The other sub-feature map is used for identity connection. A concatenation layer is then employed to combine the feature maps. The formula is as follows:

(3) $$Y=Concat(X_{1},{DWConv}_{7\times 7}(X_{2}),{DWConv}_{5\times 5}(X_{2}),{DWConv}_{3\times 3}(X_{2}))$$

where Concat means feature concatenation, and ${DWConv}_{7\times 7}$ represents a depth-wise separable convolution layer with a kernel size of 7 × 7.

Our multi-view block also includes an interaction module, which comprises both spatial purification and channel interaction modules. These modules work together to fuse features at different granularities, suppress interference, and enhance the network’s generalization ability. The output is obtained by adding the input to the enhanced feature, as described by the following equation:

(4) $$Output=X+Inter\left(Y\right)$$

where Inter represents the feature interaction operation.

### 3.2. Feature Mask Module

**Figure 4:**
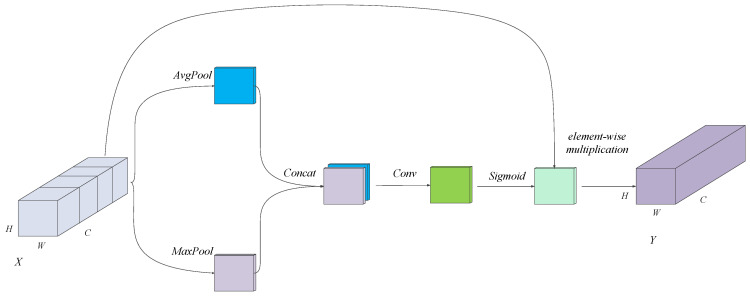
Structure of feature mask module.

Although the proposed extraction module effectively captures diverse features, certain redundant features may arise due to parallel cardinality. For instance, the features obtained through the 7 × 7 depth-wise separable convolution (dw-conv) operation may contain information already captured by the 3 × 3 and 5 × 5 dw-conv operations, along with significant background interference present in the silkworm images. To address these challenges, we introduce a simple yet effective method, referred to as the feature mask module (FMM), to purify the extracted features in the spatial domain. Figure 4 illustrates the structure of the FMM. For the input feature $X\in R^{W\times H\times C}$, extracted by our multi-order module, global average pooling and max pooling operations are first applied to summarize the spatial information, producing two one-channel feature maps. These condensed features are then concatenated to learn the spatial dependencies, which are modeled through a convolutional layer with a kernel size of 7 × 7. An activation function is then applied to suppress the pixel-wise interference, resulting in the generation of a feature mask. Finally, feature purification is achieved through element-wise multiplication between the input and the mask. The formula for the FMM is as follows:

(5) $$Y={Sigmoid(Conv}_{7\times 7}(Concat(Avgpool\left(X\right),Maxpool(X))))*X$$

where Sigmoid refers to the activation function, defined as $Sigmoid\left(x\right)=\frac{1}{1+e^{-x}}$, and “*” represents element-wise multiplication.

The FMM leverages the global average and max pooling operations to consolidate spatial information. Inspired by Xiao et al., we initially experimented with adding convolutional operations with one- and two-channel branches to aggregate more comprehensive information [32]. This approach worked effectively when the FMM was integrated into the bottleneck or multi-view modules individually. However, the performance degraded when the FMM was combined with our channel interaction module.

### 3.3. Channel Interaction Module

**Figure 5:**
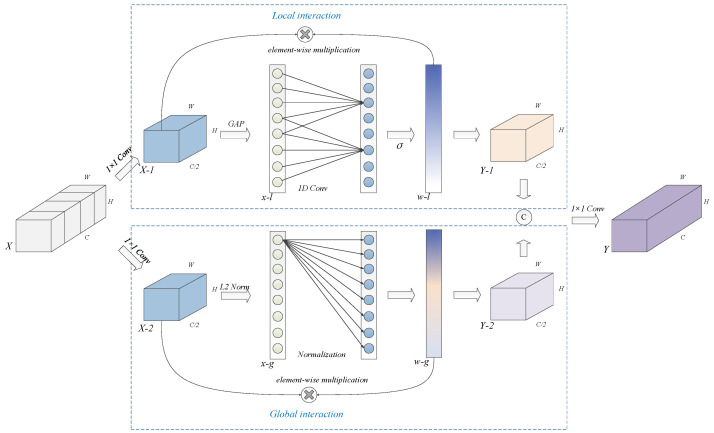
Structure of channel interaction module.

In each stage of our multi-view block, multi-order extraction and spatial purification are initially employed to learn and refine image features. Following this, feature extraction and interaction are further enhanced through stacked multi-order extraction and channel interaction modules, enabling the acquisition of more robust and discriminative representations. Figure 5 illustrates the proposed channel interaction module (CIM), which is designed to describe and recalibrate features along the channel dimension. The CIM can be conceptualized as a fusion of the channel attention mechanism [33], which facilitates local interaction, and global response normalization [34], which facilitates global interaction. During the feature interaction process, two 1 × 1 convolution layers are applied to aggregate the channels, effectively mitigating feature redundancy and yielding two distinct sub-features. The interaction process proceeds in three key steps:

(1) Feature aggregation: The local interaction employs global average pooling to condense each channel into a singular neuron, while the global branch utilizes the L2 norm to represent the channel. This operation results in two one-dimensional vectors, denoted as $X_{l}$ and $X_{g}$. The corresponding formulas are as follows:
  (6) $$X_{l}=Avgpool(X_{1})$$
  (7) $$X_{g}=L_{2}(X_{2})$$
  where Avgpool represents the global average pooling, and $L_{2}$ represents the calculation of the L2 norm.
(2) Learning the channel-wise dependencies: The local branch exploits one-dimensional convolution to capture the channel-wise dependencies, and the resulting vector is activated by the δ activation function. Meanwhile, the global branch applies global response normalization to compute the relative importance of each channel with respect to all others. The formulas are as follows:
  (8) $$W_{l}={sigmoid(1DConv}_{k}(X_{l}))$$
  (9) $$W_{g}=\frac{X_{i}}{\sum_{j=1,\ldots ,C}\left\|X_{j}\right\|}$$
  where sigmoid is the activation function. ${1DConv}_{k}$ refers to the one-dimensional convolution. k denotes the kernel size, which is determined by the number of channels, and C represents the number of channels.

(3) Channel interaction: Both the local and global branches employ element-wise multiplication to apply the learned weights to the original features. Notably, no residual connection is incorporated in the feature interaction module, as it is already utilized within the multi-order module. Reusing the residual connection could introduce confusion and weaken the network’s ability to represent features effectively. A concatenation followed by a 1 × 1 operation is employed to fuse the two recalibrated features. The formula is as follows:
  (10) $$Y={Conv}_{1\times 1}(Concat\left(Y_{l},Y_{g}\right))$$

### 3.4. WormNet Structure

Building upon the proposed modules, we introduce WormNet for silkworm ReID. 

Figure 3 illustrates the architecture of the proposed network, which follows the pipeline structure commonly seen in CNNs and Transformer-based backbones. WormNet consists of a stem, four stages of feature extraction blocks integrated with interaction modules, and a fully connected layer.

The stem module comprises a 7 × 7 convolutional layer (contained BN and ReLU layers) followed by a 3 × 3 max pooling layer with a stride of 2. The input image, with dimensions of 224 × 224 × 3, is transformed into a feature map of dimensions 56 × 56 × 64 via the stem.

Four stages of feature extraction modules are sequentially stacked to capture diverse features from silkworm images. The stage ratios are set to 3:4:6:3, which is realized by stacking the multi-order extraction and channel interaction modules. In each stage, the FMM is integrated following the first multi-order module, and the CIM is embedded after the subsequent multi-order modules. To downsample the features from Stage 1 to Stage 3, a 1 × 1 convolution layer with a stride of 2 is applied. In the final stage, the convolution stride is set to 1, resulting in a feature map of dimensions 14 × 14 × 2048. An average pooling layer is then used to obtain a one-dimensional vector for calculating the cosine similarity between the silkworm images. Finally, a fully connected layer is employed for silkworm identity classification.

### 3.5. Loss Function

In this task, the loss function consists of the triplet loss ($L_{triplet}$), the center loss ($L_{center}$), and the identity loss ($L_{ID}$).

(11) $$Loss=L_{triplet}+L_{center}+L_{ID}$$

The triplet loss is defined as follows:

(12) $$L_{tri}=max(0,d_{p}-d_{n}+\alpha )$$

where $d_{p}$ is the cosine distance between the feature vectors of the positive samples, and $d_{n}$ is the cosine distance between the negative samples. α is the margin of triplet loss, set to 0.3 [5].

The center loss is defined as follows:

(13) $$L_{center}=\frac{1}{2}\sum_{j=1}^{B}{\left\|f_{t_{j}}-c_{y_{j}}\right\|}_{2}^{2}$$

where $y_{j}$ denotes the label of the $j^{th}$ image in a mini-batch, $c_{y_{j}}$ represents the ${y_{j}}^{th}$ class center of deep features, and B is the batch size. Center loss enables characterizing the intra-class variations, and minimizing center loss increases the intra-class compactness.

The identity loss is computed as follows:

(14) $$L_{ID}=-\sum_{i=1}^{n}y_{i}\log\hat{y_{i}}$$

where n is the number of images, $y_{i}$ denotes the ground truth of the $i^{th}$ image, and $\hat{y_{i}}$ is the prediction result of the $i^{th}$ image.

## 4. Results and Discussion

### 4.1. Dataset Introduction and Experimental Settings

In this study, we introduce a silkworm ReID dataset inspired by Market1501 [35]. Under real rearing conditions, approximately 220 silkworms were reared in two separate rearing boxes. Two cameras were positioned above the boxes to capture videos from a top-down perspective, resulting in two separate video recordings. Given the silkworms’ slow movement, the videos were converted into time series images with 10 s intervals. Each silkworm was manually tracked, and the identity and location of each individual were annotated using a labeling tool (LabeImg, https://github.com/HumanSignal/labelImg, accessed on 20 June 2023). The silkworms were then extracted from the time series images based on their identity and location information, utilizing image processing techniques.

**Figure 6:**
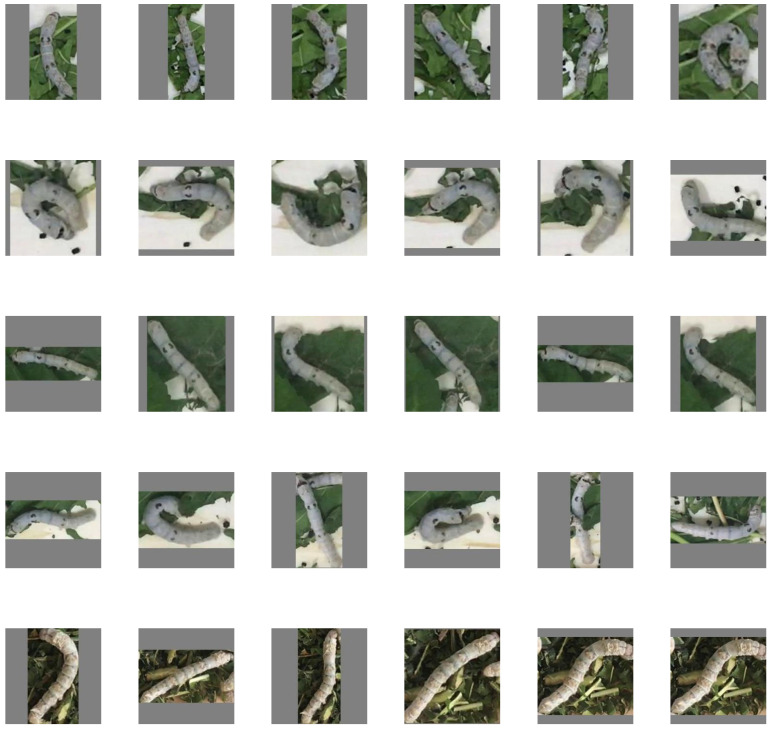
Example images of the presented dataset, where each row corresponds to the same silkworm.

As depicted in Figure 6, under real conditions, the silkworms’ postures vary, with orientations that may be parallel, perpendicular, or tilted at arbitrary angles. This variability results in silkworm images of different sizes captured in the videos. To standardize the input size, all of the images were padded with a uniform color to dimensions of 224 × 224 pixels.

The silkworm ReID dataset is constructed by randomly partitioning all images into training and test sets based on their identity numbers, with a subset of test set images designated as the query set. The number of images and individuals in the dataset are summarized in Table 1.

In this study, all of the networks were trained and evaluated using the same hardware and software configuration. The hardware included a Dell Precision 5820 workstation equipped with an Intel^®^ Core i7-9800X processor (Intel, Santa Clara, CA, USA), the CUDA-10.0 computing platform (Nvidia, Santa Clara, CA, USA), and two RTX 2080Ti GPUs (Nvidia, Santa Clara, CA, USA) with 11 GB of memory. The operating system was Windows 10 Professional (64-bit). The programming language was Python 3.7, and Visual Studio Code (https://code.visualstudio.com/, accessed on 20 June 2023) served as the development environment. The deep learning framework used was PyTorch 1.7.

All of the networks were trained from scratch with a mini-batch size of 16. The networks were trained using the training set, evaluated using the test set and query set, and the weights from the final iteration were used as the final model for network testing. To train the state-of-the-art networks in person ReID, we employed the default hyper-parameter settings and code cloned from the authors’ official repositories. For the other comparative networks, the same hyper-parameters were used, which included the following: the number of epochs was set to 50; the optimizer was Adam with a momentum of 0.9 and a weight decay of 5 × 10^−4^; and the base learning rate was 3 × 10^−4^, with a learning rate decay factor of 2, and a final decay rate of 0.1.

Two commonly used evaluation metrics—the mean average precision (mAP) and cumulative matching characteristics at rank-1, rank-5, and rank-10—were utilized to assess the performance of all of the methods. Additionally, the number of parameters, Frames Per Second (FPS), training time per epoch, and Giga Floating-point Operations Per Second (GFLOPs) were reported to evaluate the networks’ computational efficiency.

### 4.2. Comparison of Recognition Performance Between WormNet and Several Baseline Networks on the Silkworm Dataset

To evaluate the effectiveness of WormNet for silkworm ReID, we compared its performance with five baseline networks: HRNet [36], E-ELAN [31], RepVGG [37], RepVit [38], and ConvNeXt [34]. All of the models were evaluated under the same conditions.

**Figure 7:**
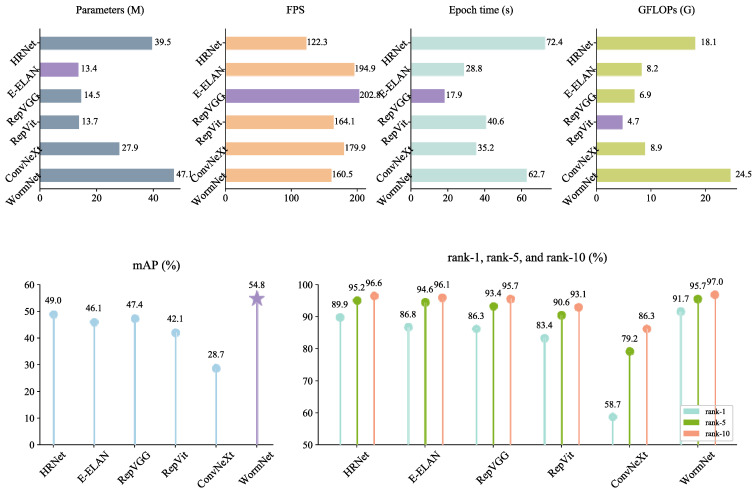
Results of comparison with baseline networks.

Figure 7 reports the results of the comparison with the baseline networks. WormNet consistently delivered the highest performance across all major metrics, achieving an mAP of 54.8%, a rank-1 of 91.7%, a rank-5 of 95.7%, and a rank-10 of 97.0%. These results underscore this network’s ability to extract and leverage a diverse range of features from silkworm images, leading to superior performance. HRNet came in second, with an mAP of 49.0% and rank-1 accuracy of 89.9%. Despite being a high-performing model, its recognition performance is lower than that of WormNet. This suggests that while HRNet can capture some key silkworm features, it may not fully address the variations in silkworm pose or background noise that are critical for high-precision ReID. E-ELAN achieved an mAP of 46.1% and rank-1 of 86.8%. Although this model shows advantages in terms of training time and the number of parameters, its lower recognition accuracy indicates that the parallel grouped computation block comes at the cost of feature richness and generalization capability in complex silkworm datasets. RepVGG demonstrated a balanced performance, with an mAP of 47.4% and rank-1 of 86.3%. While it excelled in terms of its computational efficiency, its recognition accuracy lagged behind that of WormNet. This suggests that RepVGG’s trade-off between simplicity and performance may not be optimal for the complexities of silkworm ReID. RepVit showed one of the lowest recognition rates, with an mAP of 42.1% and rank-1 of 83.4%. Its rank-5 and rank-10 also fell short, indicating that its lightweight self-attention mechanism struggles to capture the finer details necessary for this task. ConvNeXt delivered the weakest performance, with an mAP of 28.7% and rank-1 of 58.7%. This model, despite its success in other domains such as ImageNet classification, performed poorly in silkworm ReID, likely due to its inability to capture the necessary fine-grained details in silkworm images. Additionally, the large variance in the silkworm image backgrounds may have confused the network, further reducing its effectiveness.

The results of the comparison clearly show that WormNet outperforms all of the classical baseline models across all metrics. Its superior performance can be attributed to its ability to gather and integrate features from multiple perspectives, enabling it to better handle variations in silkworm orientation, background, and fine details. In contrast, the other models, which were originally designed for general-purpose image classification or object detection tasks, struggled to capture the unique characteristics required for accurate silkworm ReID.

### 4.3. Comparison of Recognition Performance Between WormNet and Feature Interaction Modules on the Silkworm Dataset

Feature interaction networks play an indispensable role in computer vision. In this section, we employed five interaction modules, including EMA [17], ECANet [33], CAA [19], SANet [21], and SCConv [23], to build recognition networks by embedding them in ResNet-50. In addition, we applied our FMM and CIM to ResNet-50, resulting in the development of a network referred to as FCNet, following the extraction purification extraction interaction strategy. We recorded and compared the performance of this network.

**Figure 8:**
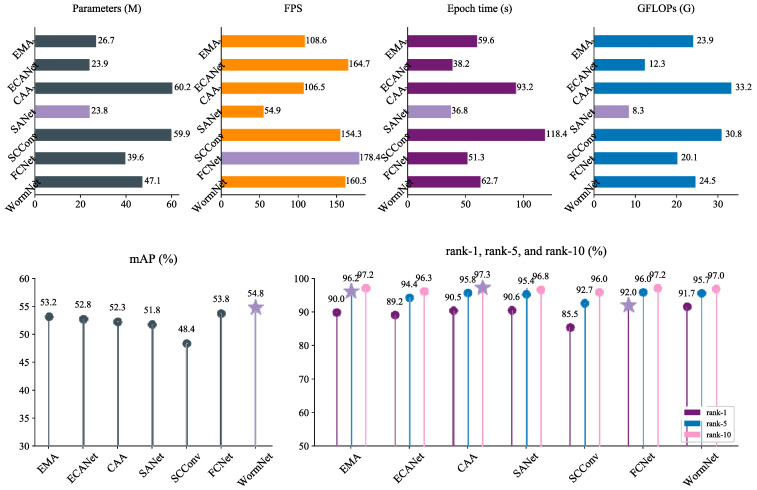
Results of comparison with feature interaction networks.

Figure 8 presents a comparison of our proposed FCNet and WormNet against five feature interaction models. Our WormNet achieved the highest mAP of 54.8%, while FCNet delivered the best rank-1 at 92.0%. These results demonstrate the superiority of our models in extracting and utilizing feature information crucial for silkworm ReID. EMA achieved an mAP of 53.2% and rank-1 of 90.0%, reflecting its solid performance in learning important feature dependencies. However, compared to our models, it falls slightly short in terms of its rank-1 accuracy, indicating a potential loss of feature relationships during its processing, which limits its overall capability in handling fine-grained silkworm details. With an mAP of 52.8% and rank-1 of 89.2%, ECANet delivered competitive results but was constrained by its focus on localized feature interactions. While it is effective in simpler tasks, its inability to capture more complex feature hierarchies limits its performance in silkworm ReID, where global feature relationships are equally critical. CAA, with an mAP of 52.3% and rank-1 of 90.5%, performed reasonably well but did not surpass our models. The results suggest that its focus on contextual dependencies was not enough to fully capture the intricate visual variations present in silkworm images, which affected its performance in more challenging cases. SANet achieved an mAP of 51.8% and rank-1 of 90.6%, while SCConv recorded values of 48.4% and 85.5%, respectively. Both models showed efficiency in computational terms, but their limited performance highlights the drawbacks of their feature processing approaches, which may have caused losses in crucial feature relationships, reducing their ability to generalize well in this task.

### 4.4. Comparison of Recognition Performance Between WormNet and State-of-the-Art Networks in Person ReID on the Silkworm Dataset

In this section, we evaluate the effectiveness of five state-of-the-art person ReID networks, ResNet-50* [5], TransReID [12], MSINet [10], CA-Jaccard [39], and OSNet [40], on the silkworm ReID task.

**Figure 9:**
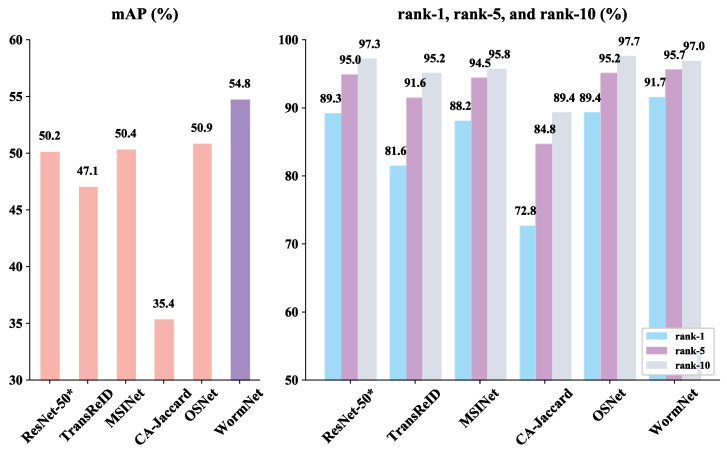
Results of comparison with state-of-the-art networks.

Figure 9 presents a comparison of the performance of WormNet alongside these models. Since person ReID networks have distinct structures and training approaches, we focus on comparing their recognition performance on the test set rather than their network properties. The results show that WormNet achieved the highest mAP and rank-1 accuracy, outperforming all other models in silkworm ReID. ResNet-50* delivered strong results, with an mAP of 50.2% and rank-1 of 89.3%, establishing it as an effective baseline for ReID tasks. The improvements in both mAP and rank-1 from WormNet indicate that its enhanced feature extraction and interaction modules significantly improve silkworm feature discrimination.

TransReID, a Transformer-based model, achieved an mAP of 47.1% and rank-1 of 81.6%. While it successfully learned diverse feature representations, it struggled to capture the fine-grained details necessary for silkworm ReID, falling short compared to WormNet.

MSINet, with an mAP of 50.4% and rank-1 of 88.2%, performed well but was limited by its reliance on standard convolution techniques. Although its multi-scale interaction improved its performance, it lacked the robustness required to handle the complexities of silkworm images, leading to a poorer performance than WormNet.

CA-Jaccard had the lowest performance, with an mAP of 35.4% and rank-1 of 72.8%. Its inability to effectively capture detailed silkworm features and its reliance on global ranking strategies, which were impacted by background interference, made it less effective in silkworm ReID.

OSNet, with an mAP of 50.9% and rank-1 of 89.4%, delivered results comparable to those of ResNet-50*, but slightly lower than those of WormNet. Its multi-scale feature fusion failed to capture the detailed nuances of silkworm images, preventing it from matching WormNet’s performance.

The above results indicate that existing person recognition networks struggle to perform highly in silkworm recognition tasks, highlighting the advantages of WormNet and the necessity of this study.

### 4.5. Ablation Analysis of WormNet

In this section, we describe an ablation analysis that was conducted to validate the effectiveness of the proposed strategies and modules. We first decompose the channel interaction module into ECANet and GRNet, so the ablation configurations include the multi-order block, ECANet, GRNet, and FMM. Next, the multi-order module is embedded into the bottleneck of ResNet-50 to build the recognition network. Then, the other modules are integrated with the multi-order block individually or simultaneously to construct the networks.

Table 2 reports the results of the ablation analysis. In particular, our multi-order feature extraction module brings significant improvements based on ResNet-50*, increasing the mAP by 3%, the rank-1 by 1.3%, and the rank-5 by 0.7%, with a similar rank-10 value, illustrating that this module enables the learning of more critical features than the bottleneck. This indicates that the multi-order module effectively overcomes the challenge of arbitrary silkworm postures in ReID tasks. The network combining multi-order and ECANet achieves the best rank-5 value, but its mAP value is weakly improved, suggesting that performing local interactions on features without spatial purification makes it difficult to enhance the network’s representation ability and improve the recognition performance. The network combining multi-order and GRNet also achieves a significant improvement in the mAP, rank-1, and rank-5 values, demonstrating that global interaction contributes to learning more important information. The network combining the multi-order and FMM network achieved a similar result to that of the single multi-order networks, indicating that spatial filtering alone, without further feature interaction, is insufficient to significantly boost the network’s recognition performance. The network incorporating multi-order, GRNet, and FMM exhibits an mAP improved by 0.8%, and a rank-1 improved by 0.8%, with similar rank-5 and rank-10 values. Although the absence of local interactions prevents this configuration from achieving optimal results, it still clearly demonstrates the effectiveness of our proposed network design strategy. The network bridging multi-order, ECANet, and GRNet increases the mAP by 0.4%, with other indicators remaining similar, illustrating that global and local interaction without spatial purification makes it difficult to improve the performance of the multi-order network. Our final structure, consisting of all of the modules, achieves the best results in terms of the mAP and rank-1. The ablation analysis validates our approach and network design for silkworm ReID.

### 4.6. Visualization of Recognition Results

In order to verify the recognition performance of the proposed WormNet, in this section, three typical images were selected from the query set, and the trained WormNet and ResNet-50* models were used to search for image sets with the same identity as the query image from the test set. For each query, the first five images found were recorded to compare the prediction performance.

**Figure 10:**
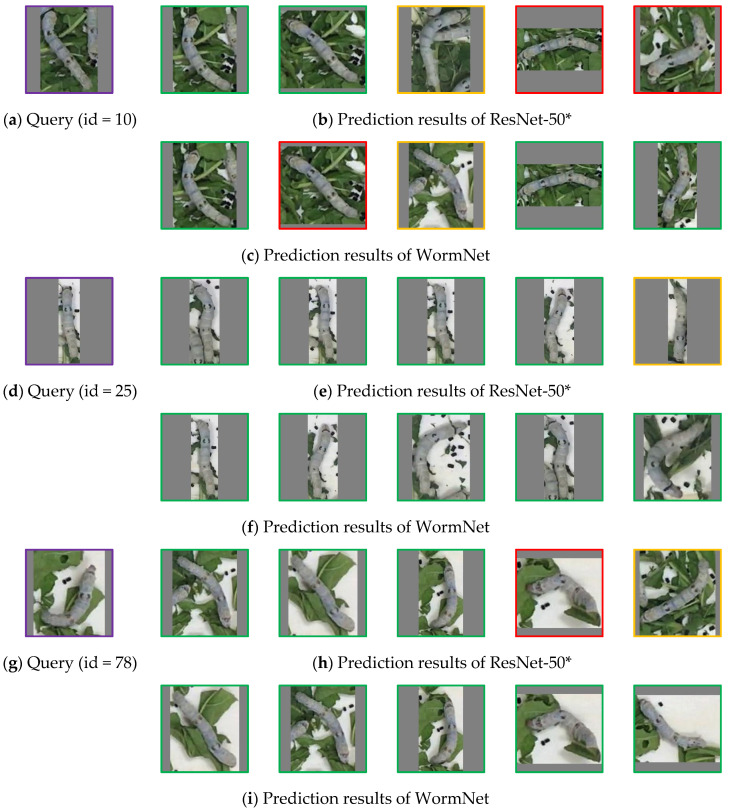
Visualization results proposed by the trained multi-view and ResNet-50* models. The image labeled with the purple box represents the query image, while the green box indicates a correct match. The orange box indicates that the image is falsely identified as having the same identity as the query image. The red box indicates that the image should have the same identity as the query image, but the model misclassified it.

Figure 10 shows the visualization result proposed by the two trained models. For the query in (a), ResNet-50* matches three incorrect images, misidentifying the third image in (b) as the same identity as the query, despite it not being the same silkworm. Additionally, although the fourth and fifth images in (b) are the same identity as the query, they are incorrectly identified as different identities. Meanwhile, for this query, WormNet also produces two misidentifications, which are the second and third images in (c). This may be due to the deep semantic differences between these two images and the substantial interference from the image backgrounds, which the network failed to accurately process in terms of local and global feature interaction. However, the fourth and fifth images in (c), despite the silkworm’s pose and orientation changing significantly compared to the query, still result in accurate recognition by WormNet.

For the query in (d), ResNet-50* incorrectly matches the fifth image in (e), as its pose is very similar to that of the query. In contrast, WormNet’s results show all correctly matched images, demonstrating its superior recognition performance. For query (g), ResNet-50* also misidentifies two images: the fourth image shows a significant pose change, and the fifth image has a more complex background, both leading to misidentification. However, WormNet maintains accurate recognition throughout.

Compared to the ResNet-50* model, our WormNet network produces more accurate query results. Meanwhile, the proposed model is able to generate accurate matches after significant changes in the silkworm’s posture; for example, Resnet-50* fails to recognize the third image in Figure 10h because its pose is clearly different from that in the query image. The visualization results demonstrate that our network has a superior search capability for silkworm images.

## 5. Conclusions and Future Work

In this study, we introduced the use of ReID into insect science, specifically for silkworm identification. We also proposed WormNet, a novel multi-view network for silkworm re-identification (ReID), designed to address the challenges of high individual similarity, arbitrary postures, and background interference. The extraction purification extraction interaction strategy, which includes a multi-order extraction block, a feature mask module, and a local global channel interaction module, demonstrated superior performance in identity recognition. Our extensive experiments show that WormNet outperforms traditional baseline architectures, feature interaction networks, and state-of-the-art person ReID methods.

The key strength of WormNet lies in its ability to accurately identify silkworms despite their high visual similarity. This makes it a promising solution for real-world applications such as pest control and ecological monitoring. Furthermore, WormNet’s robust feature extraction and interaction capabilities enable effective silkworm ReID in challenging conditions, setting it apart from other methods that struggle with fine-grained feature details.

However, several limitations remain. First, the dataset used in this study captured silkworm images at only one age, while silkworms undergo significant visual changes as they mature. Addressing cross-age identity ReID is a crucial next step. Second, WormNet’s operational efficiency needs to be improved for real-time tracking applications, especially in large-scale systems.

Future work will focus on expanding the dataset to include silkworms of multiple ages and enhancing WormNet’s efficiency in real-time applications. Additionally, we plan to integrate tracking multiple silkworms, behavior recognition, and diseased silkworms recognition to further enhance the system’s utility for monitoring silkworm populations.

Overall, WormNet offers significant advancements in silkworm ReID and provides a foundation for further research in multi-object tracking and behavioral analysis. We believe this work will benefit researchers in ecological monitoring, pest control, and related fields, helping to advance the broader field of insect ReID and behavior analysis.

## Figures and Tables

**Table 1:** Details of the proposed silkworm dataset.

Dataset	Number of Individuals	Number of Images
Training	130	4629
Testing	90	2130
Query	90	904

**Table 2:** Results of ablation analysis.

Multi-Order	ECANet	GRNet	FMM	mAP	Rank-1	Rank-5	Rank-10
× (Resnet-50*)				50.2	89.3	95.0	**97.3**
√	×	×	×	53.2	90.6	95.7	97.1
√	√	×	×	53.4	91.3	**96.5**	97.1
√	×	√	×	54.3	91.2	96.2	96.9
√	×	×	√	53.3	90.7	95.9	96.7
√	×	√	√	54.0	91.4	95.7	96.7
√	√	√	×	53.6	90.5	95.7	97.0
√	√	√	√	**54.8**	**91.7**	95.7	97.0

where “√” indicates the use of the module, while “×” indicates the module is not used. Bold values represent the best results.

## Data Availability

Data will be available from the corresponding author upon reasonable request. The data are not publicly available because they are part of an ongoing study.
